# Successful use of lidocaine hydrochloride in the management of ventricular arrhythmias in a case of pilsicainide intoxication

**DOI:** 10.1016/j.hrcr.2023.10.034

**Published:** 2023-11-10

**Authors:** Satoshi Kawada, Masafumi Tanimoto, Nobuhiko Onishi, Atsushi Takaishi, Hiroshi Morita

**Affiliations:** ∗ Department of Cardiovascular Medicine, Okayama University Graduate School of Medicine, Dentistry and Pharmaceutical Sciences, Okayama, Japan; † Department of Cardiovascular Medicine, Mitoyo General Hospital, Kagawa, Japan; ‡ Department of Cardiovascular Therapeutics, Okayama University Graduate School of Medicine, Dentistry and Pharmaceutical Sciences, Okayama, Japan

**Keywords:** Pilsicainide toxicity, Lidocaine hydrochloride, Ventricular tachycardia, Premature ventricular complex, Serum pilsicainide levels

## Introduction

According to the Vaughan-Williams classification, pilsicainide is a class Ic antiarrhythmic agent that exerts its pharmacodynamic effects via selective blockade of sodium channels; furthermore, it is mostly available in East Asia.^1^ This drug is absorbed from the gastrointestinal tract, and 75%–86% of it is excreted from the kidney.^1^ Pilsicainide is considered relatively safe and effective for the treatment of supraventricular and ventricular tachyarrhythmias without organic heart disease and is commonly used to diagnose Brugada syndrome (BrS) by inducing a coved-type electrocardiogram (ECG).^2^ However, patients with renal dysfunction and older adults are known to have life-threatening tachyarrhythmias owing to pilsicainide toxicity, even at therapeutic doses.^3^ Here, we present a case of potentially leathal ventricular tachycardia due to pilsicainide poisoning in an adult woman successfully treated with intravenous lidocaine hydrochloride and standard therapy.

**Key Teaching Points**

• Pilsicainide is a class Ic antiarrhythmic agent in the Vaughan-Williams classification, which is often used for atrial and ventricular arrhythmias in East Asia.
• Patients with renal dysfunction and older adults are known to develop pilsicainide toxicity, even at therapeutic doses.
• Intravenous lidocaine hydrochloride should be considered one of the therapies for managing pilsicainide toxicity.

## Case report

**Figure 1:**
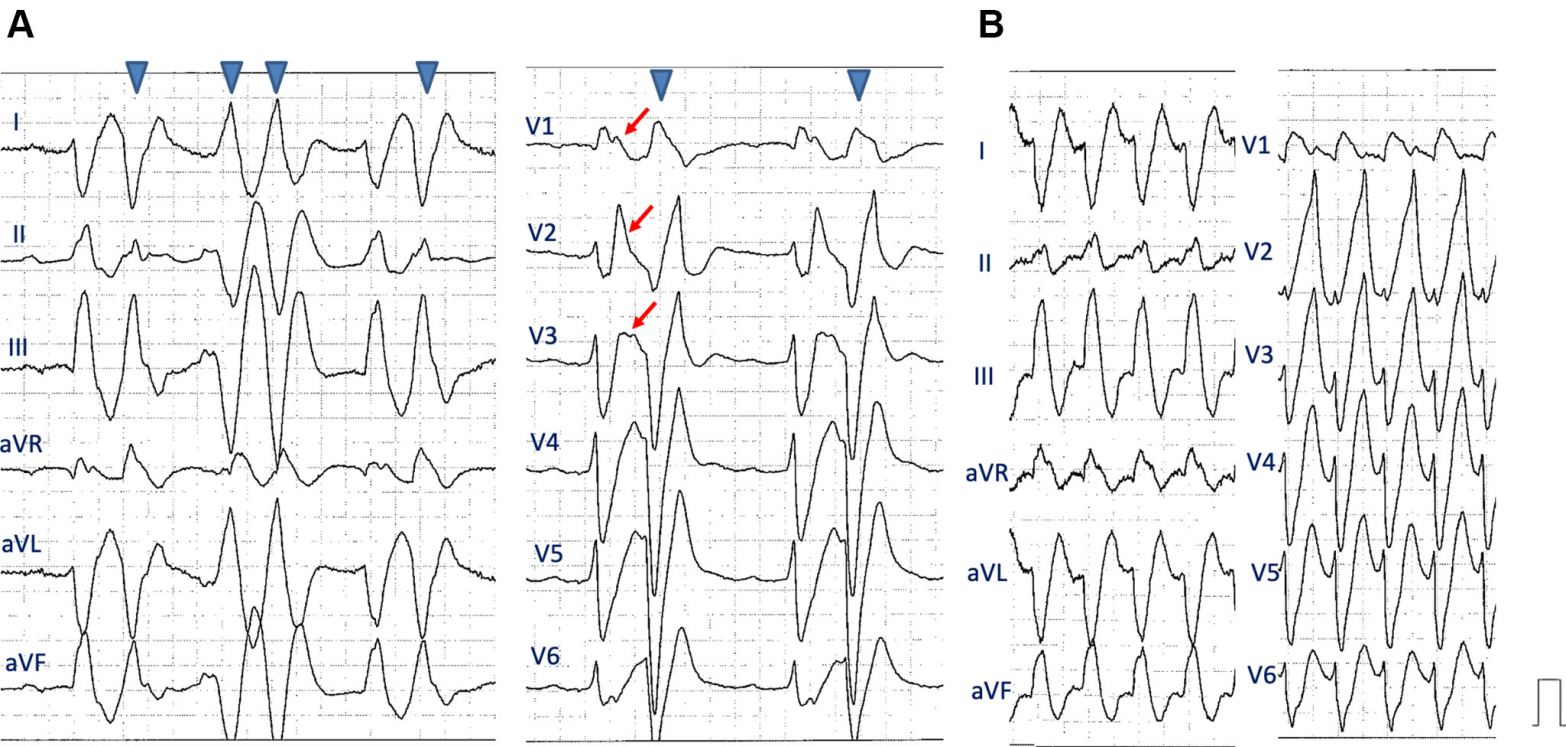
Twelve-lead electrocardiogram (ECG) upon admission. **A:** Upon admission, the ECG revealed sinus rhythm with a wide QRS complex and premature ventricular complexes (*arrowheads*). The red arrows indicate ST elevation during sinus rhythm. **B:** Sustained ventricular tachycardia occurred just before the administration of lidocaine.

**Figure 2:**
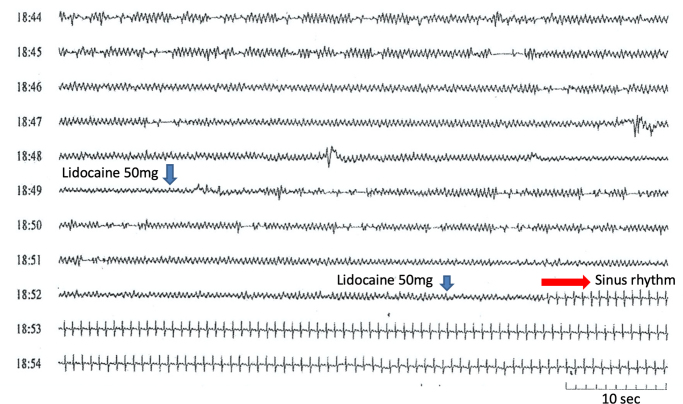
Telemetry strips before and after lidocaine hydrochloride administration. Following the administration of lidocaine hydrochloride (100 mg), ventricular tachycardia immediately ceased (*blue arrow*).

**Figure 3:**
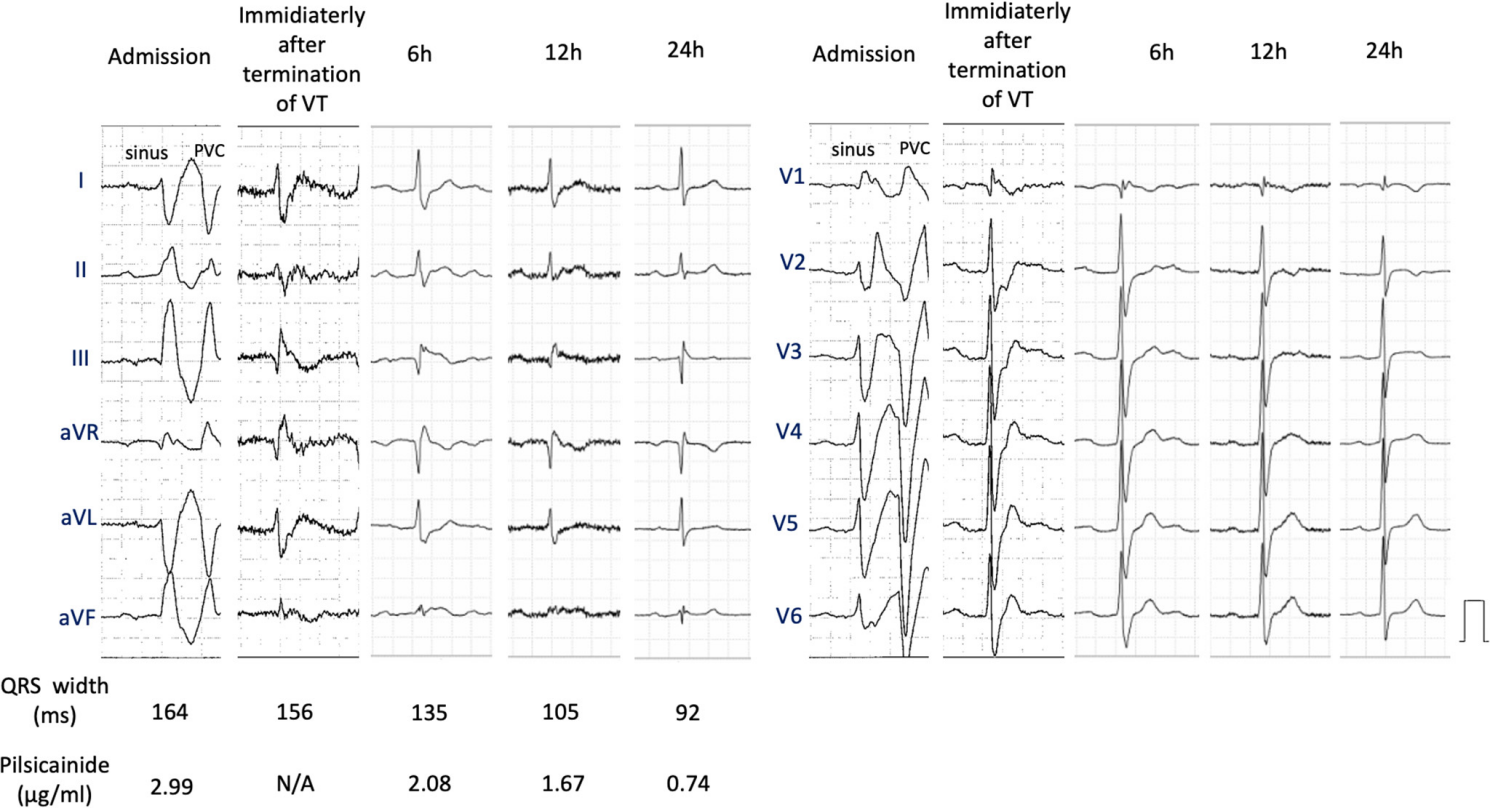
Changes in QRS width over time. As the concentration of pilsicainide decreased, the QRS width decreased. VT = ventricular tachycardia.

**Supplemental Figure 1:**
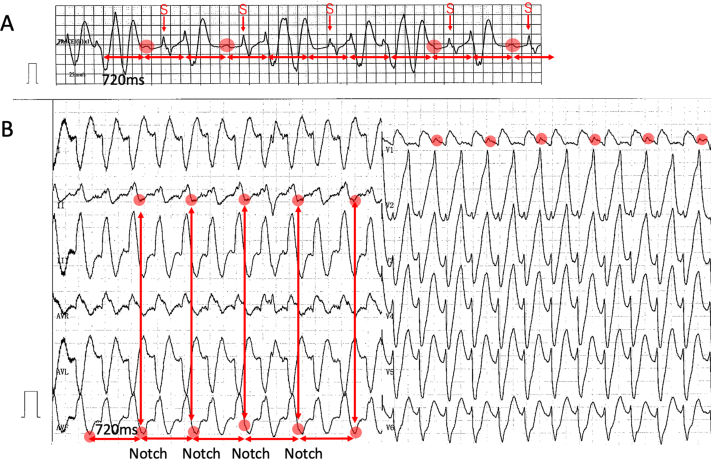
Atrioventricular dissociation during sinus rhythm with frequent premature contractions and ventricular tachycardias Sinus rhythm with frequent PVCs (A). AV dissociation was seen during VT (B). AV: atrioventricular, PVC: premature ventricular contraction, VT: ventricular tachycardia

**Supplemental Figure 2:**
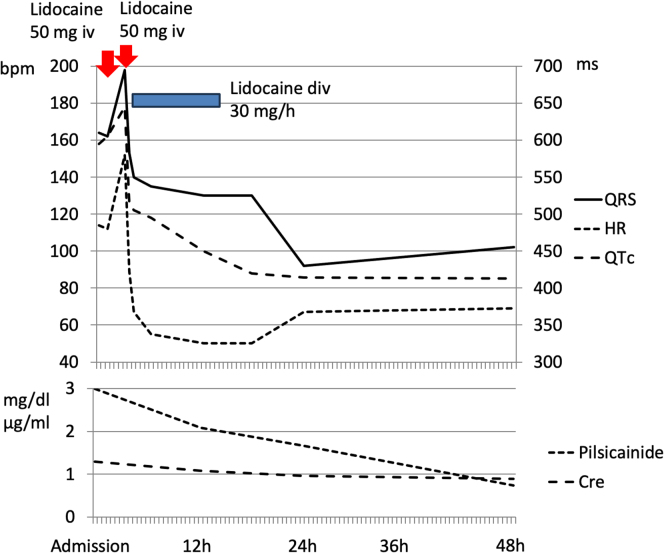
Changes in ECG parameters and pilsicainide concentration over time The red arrows represent lidocaine injections, while the blue bar indicates the continuous infusion of lidocaine for the next 12 hours. Cre: creatinine, HR: heart rate, QTc: corrected QT interval

An 84-year-old healthy woman (144.5 cm, 51.8 kg) presented to the emergency department with palpitations and dizziness via a medical service. She had been prescribed a combination of pilsicainide hydrochloride (50 mg twice a day), amlodipine besylate 2.5 mg/day, and telmisartan with hydrochlorothiazide for more than 10 years to manage paroxysmal supraventricular tachycardia (SVT) and hypertension. Upon examination, she had tachycardia (114 beats/min), normal blood pressure (136/58 mm Hg), and normal oxygen saturation (SpO2, 98%). Laboratory tests revealed normal potassium level (4.7 mmol/L) and renal dysfunction (creatinine, 1.29 mg/dL; creatinine clearance (CLcr), 23.8 mL/min). Elevated NT-proBNP levels (949 pg/mL) were observed; however, congestion and pleural effusion were not identified on chest radiography. Echocardiography revealed normal biventricular contractions without valvular heart disease. The 12-lead ECG showed sinus rhythm with a significantly wide QRS complex (QRS duration: 295 ms), ST elevation in leads V_1_–V_3_, and frequent premature ventricular complexes (Figure 1A). Based on the clinical course, we suspected pilsicainide toxicity induced by dehydration and acute renal dysfunction. Pilsicainide was discontinued, and aggressive hydration and potassium and magnesium supplementation were attempted, but the patient did not improve. We did not perform hemodialysis at that time because the effect of hemodialysis on pilsicainide toxicity is limited and controversial.^1^ After admission to the intensive care unit, the patient developed sustained ventricular tachycardia (VT) (Figure 1B). An electronic direct current shock is the most effective method and the first choice to terminate hemodynamically unstable tachycardia. However, direct current shock was not thought to solve the problem because of the repetitive form of VT. She was conscious and hemodynamically tolerated during tachycardia. Then we decided to administer lidocaine hydrochloride while preparing mechanical circulatory support with percutaneous venoarterial extracorporeal membrane oxygenation (VA-ECMO). After intravenous administration of lidocaine hydrochloride (100 mg), VT was immediately restored to sinus rhythm (Figure 2 and Supplemental Figure 1). A continuous infusion of lidocaine hydrochloride was then administered for the next 12 hours. The QRS duration just after the administration of lidocaine hydrochloride was still wide (135 ms); however, it shortened over time (Figure 3 and Supplemental Figure 2). Moreover, ST elevation in leads V_1_–V_3_ improved, no arrhythmia recurrence was observed during hospitalization, and the postadmission course was uneventful. On admission, the serum pilsicainide concentration was 2.99 μg/mL (therapeutic rage: 0.20–0.90 μg/mL), confirming the diagnosis of pilsicainide toxicity. After survival of the acute phase, 12-lead ECG was repeatedly performed during hospitalization. However, coved- and saddleback-type ST elevations were not detected, excluding BrS. Signal-averaged ECG was negative (0 out of 3). Coronary angiography was performed on day 3 after admission, revealing no significant stenosis. A previous study suggested that a positive drug challenge test did not always lead to a diagnosis of BrS.^4^ We believe the present case was a false-positive for the drug challenge test owing to pilsicainide toxicity. The patient was discharged on the 12th day of hospitalization and has not experienced any arrhythmia events for 1 year.

## Discussion

This case highlights the use of intravenous lidocaine and standard therapies for the resuscitation of a patient with hemodynamic instability owing to pilsicainide toxicity. Intravenous lidocaine should be considered to manage pilsicainide toxicity in patients with life-threatening ventricular arrhythmias.

The incidence of adverse events due to pilsicainide is 0.1%–5.0%, making it a relatively safe drug.^1^ However, patients with renal dysfunction and older adults are known to develop pilsicainide toxicity, even at therapeutic doses.^3^ Sex, age, and renal impairment (CLcr <20 mL/min) can affect systemic clearance. According to a previous report, systemic clearance is 50% lower in women than in men, regardless of body weight and CLcr.^1^ The present case involved an octogenarian woman with a small profile complicated by renal dysfunction, which could have contributed to the severity of her presentation.

**Supplemental Figure 3:**
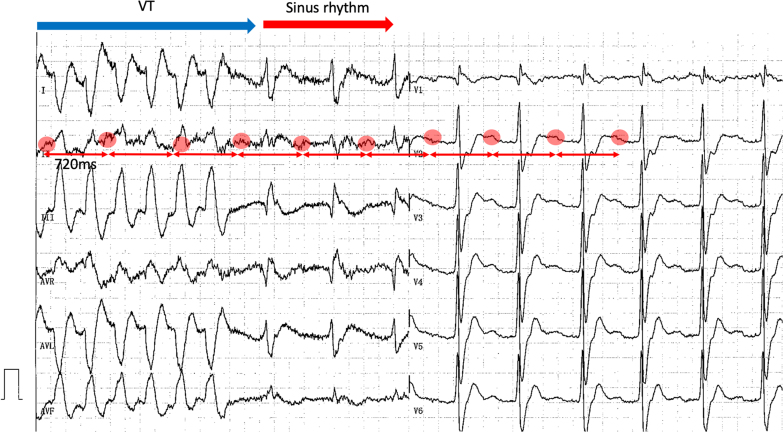
12-lead ECG immediately after termination of ventricular tachycardia The last cycle length of VT was slightly longer compared to the previous cycle length. VT: ventricular tachycardia

The correct diagnosis of VT is challenging in the present case because of the widening of the QRS interval induced by pilsicainide toxicity. Paroxysmal SVT and 2:1 atrial flutter are part of the differential diagnosis. In the present case, we could identify the presence of atrioventricular dissociation from the 12-lead ECG during tachycardia, which is a key diagnostic feature of VT (Supplemental Figures 1 and 3). The tachycardia (Figure 1B) has many similarities with the QRS complexes in sinus rhythm (Figure 1A). However, monophasic R in lead V_1_ and deep S in lead V_6_ were seen during tachycardia (Figure 1B), which supports VT rather than SVT.^5^ Moreover, the PR interval in sinus rhythm (Figure 1A) was significantly prolonged (520 ms) owing to conduction disturbance from pilsicainide toxicity. Tachycardia cycle length was 400 ms, which is inconsistent with the diagnosis of SVT in the present case.

Pilsicainide, a pure sodium channel blocker classified as a class Ic antiarrhythmic agent, delays phase 0 depolarization through high-affinity binding to activated sodium channels. The pharmacodynamic profile of this drug is characterized by slow recovery kinetics, as the onset and offset of its sodium channel-blocking action occur slowly.^1^ In contrast, lidocaine, a class Ib antiarrhythmic agent, blocks both activated and inactivated sodium channels. The dissociation of lidocaine hydrochloride occurs rapidly, resulting in minimal drug presence on channels by the end of the diastole phase. This allows for normal sodium current and conduction velocity, resulting in an unchanged QRS complex.^6^ Although the exact mechanism remains unknown, we speculate that lidocaine hydrochloride may have antagonized the effects of pilsicainide.

The general treatment and management strategies for patients with pilsicainide toxicity should be based on managing patients with sodium channel blocker toxicity. High-dose sodium bicarbonate has been reported to offset the cardiotoxic effects of this drug through serum alkalinization.^7^ Intravenous fat emulsion has been used to treat toxicity and is thought to sequester the offending agent and decrease the amount of drug available for sodium channel receptors.^7^ However, the effect of hemodialysis in patients with pilsicainide toxicity is controversial. Pilsicainide does not seem to be effectively dialyzed owing to its large volume of distribution and a slight decrease in blood levels of the drug.^8^ Furthermore, hemodialysis decreased serum pilsicainide concentrations by only 32% when evaluating pre- and postdialysis concentrations; therefore, we did not perform hemodialysis at that time.^9^ Aggressive fluid resuscitation, magnesium administration, and cardiac overdrive pacing effectively suppress tachycardia. Mechanical circulatory support, including VA-ECMO, should be considered in cases of life-threatening toxicity after the failure of medical therapy. Specifically, restoring circulation by VA-ECMO allows intrinsic drug metabolism and elimination. However, cannulation and management of VA-ECMO are often complicated, especially in patients with small profiles. Notably, lidocaine also suppresses VT induced by toxic doses of ajmaline and flecainide.^10^^,^^11^ Wynn and colleagues^11^ reported 2 cases of a proarrhythmic response to flecainide successfully treated with a 100 mg intravenous bolus injection of lidocaine. They suggested that lidocaine could be effective in similar cases of flecainide toxicity. Medina-Ravell and colleagues^10^ reported 2 patients in whom VT developed with small doses of ajmaline. One of them was successfully treated with a 100 mg intravenous bolus injection of lidocaine.^10^ The present case also suggests that lidocaine can be used as a therapy for the management of pilsicainide toxicity.

Flecainide, ajmaline, and pilsicainide are frequently used to diagnose BrS unmasked by type 1 ECG.^2^ However, life-threatening ventricular arrhythmias occur in approximately 2% of patients during drug challenge tests and sometimes require VA-ECMO during an arrhythmic storm.^12^ ^13^ ST elevation in leads V_1_–V_3_ was observed in the present case during pilsicainide intoxication, and toxic doses of pilsicainide frequently induced ST elevation, similar to the Brugada ECG.^14^ Lidocaine does not aggravate ST elevation or VT in patients with BrS.^15^ Observing the present case could also provide a clue for treating life-threatening arrhythmias during drug challenge tests in patients with BrS.

## Conclusion

This case report suggests that the injection of lidocaine hydrochloride should be considered as a therapy for managing pilsicainide toxicity with life-threatening ventricular arrhythmias. Invasive therapies, including transcutaneous pacing, hemodialysis, and VA-ECMO, should also be considered for patients who are refractory to standard medical therapies.
